# An “integration” of professional identity formation among rural physicians experiencing an interplay between their professional and personal identities

**DOI:** 10.1007/s10459-024-10337-z

**Published:** 2024-05-13

**Authors:** Junichiro Miyachi, Miho Iwakuma, Hiroshi Nishigori

**Affiliations:** 1 Center for Medical Education, Graduate School of Medicine, Nagoya University, 65 Tsurumai-cho Showa-ku Nagoya, Aichi, 466-8560 Japan; 2 Academic and Research Centre, The Hokkaido Centre for Family Medicine, Hokkaido, Japan; 3 Department of Medical Communication, School of Public Health, Graduate School of Medicine, Kyoto University, Kyoto, Japan

**Keywords:** Professional identity formation, Rural physicians, Integration, Identity narratives

## Abstract

The present understanding of professional identity formation is problematic since it underrepresents minority physicians and potentially excludes their professional identity formation experiences. Rural physicians are expected to have similar underrepresented aspects as minority physicians because of their specific sociocultural contexts and consequent private–professional intersection, which lead to ethical complexities. Therefore, to bridge this research gap, we interviewed 12 early- to mid-career Japanese physicians working in rural areas and explored their experiences. Through a narrative analysis guided by Figured Worlds theory, we analysed the data by focusing on the vocabulary, expressions, and metaphors participants used to describe their experiences. A central theme emerged concerning how the rural physicians configurated their personal versus professional participation in their local communities. Further, their identity narratives varied regarding how they constructed their identities, rural communities, and relationships as well as their identity formation ideals and strategies to achieve them. Informed by ‘Big Questions’ concerning worldview framework, we delineated four identity narratives as prototypes to describe how they participated in their communities. These identity narratives provide a preliminary understanding of how diverse identity formation is for rural physicians. In addition, our findings exposed the current professional identity formation framework as potentially biased towards single forms of participation in monolithic communities, overlooking complicated forms of participation in multiple communities. We argue that applying frameworks and concepts to capture these multiple forms of participation as well as revisiting the ‘discourse of integration’ are necessary steps to overcome the limitation of the current understanding of professional identity formation.

**Supplementary Information:**

The online version contains supplementary material available at 10.1007/s10459-024-10337-z.

## Introduction

Professional identity formation (PIF) is widely accepted as a key process in the development of a physician and an integral part of developing and delivering medical education curriculum (Cruess et al., 2015; Jarvis-Selinger et al., 2012). Existing literature describes the conceptual representation of PIF as a developmental process that entails two aspects: individual psychological development and the socialisation of the person into roles and forms of participation in a particular community (Jarvis-Selinger et al., 2012). However, recently, it has been suggested that empirical research on PIF is biased because it ignores wider historical, cultural, and social contexts (Volpe et al., 2019).

Consequently, owing to the underrepresentation of minority physicians and their PIF experiences, the present understanding of PIF is limited (Wyatt et al., 2020; Wyatt, Balmer, Wyatt et al., 2021a). This exclusion was recently confirmed by an empirical study (Wyatt, Rockich-Winston, et al., 2021) of Black/African American physicians’ PIF experiences. The study illustrated how context as well as physician and personal identities are interconnected, and that focusing primarily on clinical and pre-clinical experiences by PIF researchers may neglect this interconnectedness.

Rural physicians’ PIF experiences are expected to include similar underrepresented aspects to those of minority physicians because of their specific sociocultural contexts. This is because rural physicians usually live in and need to be members of their local rural communities, thus they have to be prepared for rural core values and the local community’s way of life (Woloschuk et al., 2005). Given the small population that typically characterises rural communities, inevitably, neighbours and friends with whom rural physicians have ‘non-clinical’ interactions (i.e. personal interactions out of the office) could also be their patients (Gingerich et al., 2021; Scopelliti et al., 2004; Zur, 2006). In such interactions, clinical requests by community members made outside the office based on personal relationships and friendships may sometimes conflict with how the physician responds, as this response may be based on internalised norms of the medical profession (Thach et al., 2018). This conflict causes rural physicians to encounter problems with managing dual relationships and setting appropriate boundaries. They have to deal with the paradoxical and complex task of adhering to urban-based professional community norms while respecting the rural community’s core values and way of life (Gingerich et al., 2021). Existing research has clarified that this ethical complexity engenders rural-specific strategies (Gingerich et al., 2021) and internalised norms (Brooks et al., 2012). However, how this ethical uniqueness based on the specific sociocultural contexts of rural practice influences PIF and the consequent need to navigate various roles and circumstances remain under-studied topics (Crampton & Afzali, 2021). This may be because the current PIF perspective fails to elucidate such unique experiences; the primary focus on clinical experiences overlooks the influence of wider non-clinical experiences on rural physicians’ PIF. Therefore, this study describes rural physicians’ PIF by focusing on their experiences outside of their clinical practice. By doing so, it elucidates unexplored aspects of rural physicians’ PIF, which will be practically useful in supporting the successful recruitment and retention of rural physicians while theoretically contributing to progressing and ensuring an inclusive understanding of PIF.

## Methods

### Ethical approval

This study was conducted with the approval of the Ethics Committee of Kyoto University.

### Study design and theoretical orientation

Given that the current study’s subject of interest lies at the intersection of socio-cultural contexts and an individual meaning making process, this study was based on a social constructivist paradigm ^28^. We adopted a socio-cultural approach to explore PIF. This approach entails considering identity as a social construction of the self that involves persuading others (and oneself) of who one is and what one values, positioning oneself in the world, and relating to others through reflection and observation (Penuel & Wertsch, 1995).

Our methodology was guided by narrative analysis, which is appropriate for researching experiences over time (Bleakley, 2005). The narrative of a physician’s experience is a platform for investigating their identity formation in two folds (Clandinin et al., 2017). First, their narrative provides a probe into their prior experiences and reflections. The contents of a story of interaction with patients outside of clinical settings provide the narrator’s reflection on their PIF in the context of their rural placement. Second, their act of narrating is a performative process through which they position their own and others’ identities against the audience (Monrouxe & Rees, 2015). That is, the interview is regarded as an ongoing process of constructing their identities by positioning themselves in the act of telling their stories to researchers (Monrouxe, 2010).

We did not rely on any predetermined theoretical frameworks at the beginning of this study, since we believed that their inclusion might obscure certain unexposed aspects of the identity narratives of rural physicians. Instead, we gradually identified appropriate theoretical frameworks during data collection and inductive data analysis, as detailed in the data analysis section. Ultimately, we used Holland et al.’s (1998) Figured Worlds theory and Taves et al.’s (2018) ‘Big Questions’ that articulate worldviews as theoretical frameworks.

### Study context and participants

The study was conducted from 2015 to 2017 in Japan. Interview participants were initially recruited via a snowball sampling technique, targeting Japanese physicians who worked or had worked in rural areas for 1−10 years. Two main groups of physicians met these criteria. The first constituted graduates of Jichi Medical University, a university that requires all graduates to work as a doctor in rural areas for seven years from their third to the ninth year, excluding a 1–2-year elective period (Inoue et al., 1997). The second comprised trainees or graduates of the general practitioner program by the Japan Primary Care Association (Kato et al., 2019). Some accredited programs in this scheme have a rural placement program during the training period, so some of the trainees obtained rural experience during their training while others continued their rural work even afterwards. As the study progressed, we purposively sampled interviewees to include varied geographical rural regions, family, and gender backgrounds to obtain diverse data. Eventually, 12 participants were included in the study. The participant characteristics are shown in Table 1.

**Table 1:** Participant characteristics

Pseudonym	Age	Gender	Specialty (at the time of interview or prospective)	Duration of rural practice (years)	Duty or not	PGY
Akari	30s	F	FM	1	N	12
Bakuto	30s	M	FM	4	N	8
Chizuo	30s	M	FM	7	N	11
Daisuke	30s	M	FM	3	N	8
Enishi	30s	M	FM	7	N	12
Fumitaka	30s	M	Psychiatry	3	Y	7
Gekka	30s	F	Anaesthesiology, FM	1	N	14
Hayato	30s	M	FM	3	Y	6
Ichiro	30s	M	FM	2	Y	5
Junko	50s	F	FM	5	N	13
Keisuke	20s	M	Cardiology	1	Y	5
Leona	30s	F	FM	2	Y	6

PGY: postgraduate years, FM: Family Medicine

### Data collection

We collected data using a semi-structured interview schedule. The first author asked the participants to talk about their upbringing, how they came to commence rural practice, episodes of interactions with patients in non-clinical settings including difficulties in understanding or responding to the community’s expectations and norms, and to reflect on their perceptions and responses. In addition, participants were asked about the norms and attitudes they hold as rural doctors and their views on living within the community. The interview form is shown separately (Appendix 1). Interview data were digitally recorded and transcribed verbatim.

### Data analysis

Data analysis was an iterative process that comprised data-driven inductive analysis, reflexive identification of theoretical frameworks, and theory-guided analysis. Our initial analysis was data-driven and guided by narrative analysis based on the sociocultural approach to identity formation. During the early phase of the study, the first author (JM) initially familiarised himself with the data by reading and re-reading the transcripts. Next, he coded the interview data, gradually constructed codes, and recorded his tentative interpretations regarding the stories each participant had constructed. Subsequently, the second (MI) and last author (HN) reviewed the transcripts and coded the data to critically examine the first author’s interpretations. We then discussed our findings, including the features of identity narratives used by each participant, and explored theories that could guide our study in delineating the divergences of rural physicians’ identity narratives. This exploration was a circular rather than a linear process and included identifying a provisional guiding theory, reinterpreting data, examining whether the theory fully articulated the diversity of identity narratives, and exploring alternative theories.

In the early stage of the study, inductive data analysis led us to tentatively interpret participants’ narratives as identity formation through cross-cultural interaction between participants’ own culture and that of the rural community. Accordingly, we attempted to apply a cross-cultural perspective such as acculturation theory (Sam & Berry, 2010) to guide our analysis. However, as the study progressed, we noticed that contrasting physicians’ own ‘culture’ and that of the rural community was only one way of constructing and configuring individual actors such as oneself, other people, and the rural area. Through the reflexive process, we concluded that it was necessary to explore an alternative theoretical framework able to capture the diverse ways of constructing actors and their configuration in a narrative form. We ultimately applied Holland et al.’s (1998) Figured Worlds theory and Taves et al.’s (2018) ‘Big Questions’ used to analyse worldviews.

The concept of ‘figured worlds’, proposed by Holland et al. (1998), describes a realm of interpretation shaped by social and cultural constructions where characters are recognised, certain acts are accorded significance, and particular outcomes are valued over others. These worlds provide the backdrop against which social positions are named and the loci in which identities are formed. Identity within this framework is seen as a dynamic process, continually shaped through constructing worlds and ‘self-authoring’ or self-narration and interaction with these constructed worlds. Our study leverages this theory to explore how identity is crafted, focusing on the narratives of rural physicians outside clinical settings. This approach allows us to examine the construction of the self and the social world through our participants’ stories, highlighting the iteration between sociocultural contexts and identity formation.

To capture the diversity of participants’ identity narratives, we focused on delineating their construction of the self and the narrativized features of ‘rural community’, recognizing that these constructions vary among participants. This led us to adopt Taves et al.’s (2018) ‘Big Questions’ framework, which examines worldviews through multidisciplinary lenses, encouraging reflection on the fundamental aspects of ontology, epistemology, axiology, and praxeology. We adapted this framework to explore the construction of identity in rural settings, with revised questions tailored to our study’s context guiding our analysis: describing the rural community (ontology), knowing who oneself is within it (epistemology), describing ideal identity models and underlying values (axiology), and determining the best strategies for achieving these ideals (praxeology). This approach provided a structured way to articulate the varied identity narratives among our participants.

Based on these theoretical frameworks, we revisited the data. JM focused on the units of experiences or events and interpreted what languages and forms were applied in the context. Particularly, he analysed what vocabulary and expressions − including metaphors − were employed to portray individuals, materials, their environment, and broader rural areas. JM explored how these descriptions were inter-related and contextualised in the events and experiences. This analysis extended to examining how individual experiences were woven into the broader tapestry of each participant’s whole data. Hiroshi Nishigori (HN) and Miho Iwakuma (MI) rigorously reviewed the validity of the analysis until they reached a consensus. Then, JM compared and contrasted each participant’s analysis to identify common structures in the identity narrative while also highlighting unique aspects that distinguish each identity narrative. Recognising that each identity narrative reflects a distinct worldview, we chose to present identity narratives as prototypes rather than as elements or fragments. These prototypes encapsulate descriptions of the self and the rural community, the construction of identity, ideals of identity formation with their underlying norms, and the strategies deemed essential to achieve these ideals. Throughout this process, all of the researchers assessed the validity of the analysis and modified each prototype whenever necessary. The entire analysis was conducted in Japanese and finally translated into English.

### Reflexivity

The researcher team consisted of JM, a family physician and medical education researcher with experience practising in multiple Japanese rural areas; HN, a general internal physician and medical education researcher with some rural experience; and MI, a non-medical expert in medical communication research. JM and HN’s background, while a unique resource for analysing the data, was also recognised as a potential limitation that could skew the analysis. Both JM and HN have been involved with clinical and educational tasks, which put them under the influence of the current PIF perspective. This fact makes it difficult, if not impossible, for them to uncover underrepresented aspects of rural physicians’ PIF. However, MI’s expertise in social sciences and their lack of a medical background provided a contrasting perspective, helping to identify and challenge the unacknowledged assumptions made by JM and HN.

To make use of these unique perspectives and enhance reflexivity, all the researchers had a reflective conversation, including how their prior experiences affected the analysis. JM and HN actively disclosed their prior experiences regarding the topics during the analysis, and HN, as a more experienced researcher, attempted to point out JM’s rural experiences underlying the interpretation by sharing his alternative interpretation of the data. Further, MI attempted to contrast the interpretations and identify the implicit, unrealised assumptions that seemed to underlie both JM and HN’s experiences to enhance reflexivity and deepen the analysis. For instance, a participant’s description of receiving clinical requests outside formal settings elicited different responses from JM and HN. JM felt awkward when faced with clinical requests made outside the office, while HN welcomed them. Probing this difference further, MI interrogated what the word ‘outside’ meant for JM and HN as well as the participants, revealing varied perceptions related to ‘outside’ experiences. This approach facilitated us to develop a more nuanced understanding of the diversity of identity narratives.

## Results

Participants used various identity narratives to describe and configurate their non-clinical interaction experiences with their patients. Each narrative had a whole structure that was underpinned by assumptions regarding how rural communities, one’s identity, and their configuration were constructed. We identified two structures: binary oppositive and multi-faceted structures.

The binary oppositive structure is based on a contrast between the physician as the ‘self’ and the rural community members as ‘others’. The local community is regarded as reflecting totalised unit and is configurated as opposing to physicians. In this ‘black-or-white’ perspective, identity formation is represented as one’s positioning and movement between the bipolar extremes of ‘physician identity’ and ‘local member identity’.

The multi-faceted structure is based on considering identity as a dynamic relational process that emerges through each particular interaction among wider relationships. In this perspective, identity is a facet arising from relationships between rural physicians and local members. Because of the overlapping features of relationships in rural communities, one’s identity becomes multi-faceted, and physician identity becomes only a part of this identity. In turn, the local community is not constructed as a single unit, but regarded as a web of relationships. The multi-facetedness of identity leads to fluidity since identity is transitory from one facet to another depending on the interactions and contexts of their everyday life. Identity formation is a relational practice amid the fluidity. This multi-facetedness of identity and its fluidity, along with the diverse nature of local community relationships, constitute the core of the structure.

These features were reflected in how each narrative demarcated inside and outside clinical settings. In the binary oppositive narrative, the boundary was set physically (e.g. inside examination rooms) and chronologically (e.g. clinical hours). Contrastingly, in the multi-faceted narrative, the boundaries between a situation where clinical discourse is open, cautiously exchanged, or totally excluded were socially constructed by the interplay between physicians, patients, and their circumstances.

Based on these structures, an overarching theme pertaining to the narratives was how the participating physicians constructed their identities in the wider rural community. Each narrative was oriented by its specific ideals and the strategies required to achieve them. Focusing on their variations, we identified four types of identity narratives that represent different processes and features of rural physicians’ PIF; an overview is shown in Table 2. Some participants consistently used only one identity narrative, while others used more than one narrative depending on the period of the experiences they narrated. The following subsections illustrate the specific features of each identity narrative accompanied by relevant data. This is followed by a brief description of how some participants used multiple identity narratives. All participant names appearing below are pseudonyms.

**Table 2:** Overview of identity narratives and their underpinning structures

Identity narratives	(i) Policing one’s distance from ‘the locals’	(ii) Struggling to become one of ‘the locals’	(iii) Fixing identity among ambiguities	(iv) Maintaining multi-faceted identities for flexibility
Underpinning structure	Binary oppositive narratives		Multi-faceted narratives	
How is the rural community described?	A totalised unit	A totalised unit	A web of multiple relationships	A web of multiple relationships
How is one’s identity constructed in the rural community?	Encapsulated as a quantitative distance from the local community	Emplaced between the immature ‘physician identity’ and the mature ‘local member identity’	Formed and arose through daily interactions and activities in the context of particular relationships	Formed and arose through daily interactions and activities in the context of particular relationships
What is the ideal identity formation perspective?	Keep the distinction between physician identity and personal identity Maintain some distance from the local community	Approach and construct an equivalent identity to that of the locals	Reconcile identities and particular situations within a web of relationships	Maintain flexibility and generativity concerning one’s identity formation to cope with everlasting negotiated processes in the rural community
Strategies to achieve or maintain the ideal identity formation perspective	Separation Control	Minimisation (e.g. partialisation, localisation, and emphasis of their individual aspect)	Arrangement Specification	Liminalisation Polyphonisation
To what values and norms underlying ideal identity formation is the physician oriented?	Oriented to detachment, physician perspective (e.g. objectivity)	Oriented to the norms of the local community (e.g. reciprocity)	Oriented to a range of professional responsibilities (e.g. quality of care, confidentiality, access to care)	Oriented to sustainability

### Policing the distance from ‘the locals’

The first identity narrative was characterised by a process through which the participant kept their physician identity separated from the local residents. The identity ideal here was to maintain the separation between personal and professional identities and maintain a differentiated physician identity. These participants avoided interactions with patients outside clinical settings whenever possible or carefully monitored and minimised them if inevitable (due to geographical overlap). This avoidance of interaction was not only represented as a personal preference but was also justified as a requirement for a physician to provide appropriate medical care. For example, as part of his reflections regarding frequent encounters with patients in non-clinical situations, Chizuo stated:

It was really unpleasant since I had no personal time. I always had to be careful and manage what I was doing […] I thought it was better to be distant since I could provide […] objective medical treatment based solely on medical judgement.

This ‘distant’ and ‘objective’ physician persona was a typical icon representing the norms that participants used to justify this type of identity narrative. Reflecting the narrators’ adherence to the ideal and underlying norms, two layers of differences were emphasised in their storytelling: the distinction between their personal and professional identities and the distance between their ‘personal’ identity and the local community. The creation of these two layers of distance, or ‘separation’, is a strategy that emerges in how the participants constructed their identities through narrating. For example, Dr Fumitaka drew a distinct line between his identity formation and that of his seniors who had lived a mixture of two identities: a private individual and a medical professional. He stated this as follows:

From other graduates, I had often heard about their interactions with their local community [members], such as going to a community festival. But there was a line between their ways and mine. I didn’t have a sense of being a local member. Rather, I worked in the community as a despatched doctor. My personal time was exclusively for myself and I didn’t intend to immerse myself in this community. I wanted to contribute to medical care in this area, but I did not do so as a local member… I didn’t care who was the mayor, how the economy went, [or] whether the young population would increase or decrease in Minaduki-town.

This doctor constructed himself as an outsider using the description of a ‘dispatched doctor’, essentially distancing himself from being a rooted physician and drawing a strict distinction between his personal and physician identities. By confining his contact with the local community to healthcare activities, he constructed the distance from the local community in his storytelling.

However, because of the small populations that typically characterise rural communities, the likelihood of the physicians encountering patients in their private lives cannot be dismissed. Some participants attempted to minimise such inevitable interactions in their identity formation in ways that are aligned with the aforementioned ideals and norms. For example, Bakuto described how he would prefer to manage clinical interactions when community members are involved in his private life:

Bakuto: I feel it would be appropriate if I could deal with all people equally, but relationships with patients fluctuate. They have ups and downs. […] If I maintain contact with a patient for a long time through home visits, the relationship with her grows and I [tend to] get along well with that patient; however, I feel awkward [when I] see some patients whose clinical management sessions didn’t go well. Such fluctuations happen. So, I have got no choice but to be aware of such fluctuations […].

JM: What do you mean by ‘equally’?

Bakuto: I mean, I hope patients wouldn’t depend on me. If I leave and other physicians come to this town, I hope people will not compare them and me. Or, it’s not good if someone says ‘I prefer this doctor among physicians in this clinic’.

Unavoidable interactions with patients outside the clinical settings were positioned as ‘variations’ to be monitored and minimised. This act of ‘control’ is another specific identity formation strategy in this identity narrative. Doctors regard identity is encapsulated as a measurable distance between oneself and the local community, particularly when that community is considered a homogenous unit. The necessity of controlling this distance is particularly emphasised in the out-of-clinical-hours or outside-health-institutions narrative. Interactions spatiotemporally ‘outside’ the clinical setting required continuous adjustment through separation and control to establish the norm of being an ‘objective and detached’ physician.

### Struggling to become one of ‘the locals’

The second identity narrative could be understood as a quest to achieve an identity comparable to that of local members. The participants described that ideal identity development entails abandoning the physician identity and adopting the local community identity. Being fixated on the physician identity was a sign of immaturity and ‘shaking off’ the physician identity was necessary to immerse oneself in the local community successfully. This was illustrated by Leona:

My perspective on being immersed into the local community was that I could interact with the locals in person without the ‘doctor’ aspect. Wherever I went [out], I was treated as a doctor. I wanted to throw away a kind of ‘mask’ of being a physician, but I couldn’t. I thought it’s because I hadn’t immersed [myself] in the community enough.

The physicians described their personal identities as facets to be connected with the local community, as shown by Hayato’s explanation regarding his personal identity:

When someone says ‘[this is] what I can do as a physician’, maybe he has excluded his personal aspect. But what I mentioned as ‘I will consider what I can do as an individual’ is based on an assumption that I place my personal aspect within the local community and [that] I live there and then […]. The starting point is the same for everyone. Next, I [consider that I] happen to be a person with a special skill − medical care. Then, I explore what I can do [with that], something related to medical work.

Accordingly, the participants described interactions with patients in non-clinical settings as an opportunity to progress their unfinished identity formation through participating in local activities and forming relationships as members of the community. This progression is described by Hayato:

You would go around and talk with locals. You would drink or eat with them. That’s like living under the same roof. I’ve often participated in the local festivals and found out [that] some [of the] guys working at the food stands were my patients. Such a process had gradually formed mutual recognition. A feeling that I’m involved in Nagatsuki town is established through such recognition from other people. Recognition from others makes me become Nagatsuki town’s physician. Other people said to me you are a ‘guy’ in Nagatsuki town rather than a local physician, so I became a guy in Nagatsuki town. My feeling is like that.

This placement of personal identity in the local community affected how they described their professional identities. The research participants partialised (‘a person with a special skill’) or localised (‘Nagatsuki town’s physician’) their physician identity to distance themselves from the general image of ‘doctors’. The ‘shaking off’, or *minimisation*, of their physician identity is a characteristic strategy to emphasise one’s proximity to ‘the locals’. Hayato described the process of shifting his identity from an ‘urban doctor dispatched from Nagatsuki city’ to a ‘local member’ over time as follows:

Becoming a physician of Nagatsuki hospital was a process that [involved me having] a more and more personalised relationship with my patients, and some of them say they want to be seen by me instead of other doctors. Being seen as an ordinary person was progressed by interactions in which locals brought me treats such as sandwiches or Japanese noodles [that] they made. Such interactions outside the office made me an ordinary person in the local community.

The way the participants justify their practices was also distinctive. They emphasised various behaviours including that their clinical practices were based on the ‘ethics’ of their local community rather than medical professionalism. For example, Ichiro positioned medical advice in non-clinical settings as a normative practice by juxtaposing medicine with a non-medical job (i.e. being a farmer).

I’m a medically skilled person here. A farmer who knows a lot would surely be asked for advice about rice paddies when they go out drinking. It is something like that for me. […] So, I have accepted the fact that I will be asked medical advice since I’m a local resident with medical care skills.

He mentioned the importance of performing the duties that doctors are usually exempted from in the community (e.g. participating in events and contracting out as officers of the community association) to gain an equivalent status with the local people.

My perspective is that rural physicians are like migrants who are allowed to live in the village. This is different from the attitude that rural physicians move there for the locals. Rather, we are welcomed by the community. In return, we have to contribute to the community by some means. Without it, we cannot become a member of the community.

However, even if rural doctors try to minimise their physician identity, they continue to find themselves being considered only as physicians and being treated differently from the public. Because of this persisting feature of the physician identity, in this narrative, they sometimes described the ideal identity goal as unattainable. The following narrative by Hayato described how he struggles with and strives for this unattainable identity formation ideal.

When someone passes away, I am sad, but I feel embarrassed that I am no longer as confused about their demise as before. As a member of the local community, I think I should be grieved and upset when a neighbour dies, but my inner physician draws a line at some point. The more the years go by, the less upset I get when my patients die. This is something that I’m still struggling with. […] Last year, my grandmother passed away unexpectedly. […] When my grandmother passed away, I was so upset and wept so much, and I realised that my sentiment towards my neighbours is somewhat different from [those towards] my family members. I wonder [if] it is because I’m a physician in this community or my involvement with the local people is just a bit different.

### Fixing identity among ambiguities

The third narrative represents a continuous on-the-spot fixing of identities amidst the fluidity derived from the multi-facetedness of identity. The experience of interacting with patients was constructed as a situation where the fluidity of identity arising from multi-faceted identities caused ambiguity. The identity narrative focused on the process of exploring and finding one’s identity to solve this ambiguity in a context-specific relationship. This construction of the fluidity as an ambiguity, that is, a problem needing resolution, and the consequent motive to resolve it, was an integral element of this identity narrative. For example, Leona gave the following example of a situation where an older adult she met asked her if she should see a doctor about her grandchild’s runny nose when she was picking up her own child from day-care.

When an old lady asked me, ‘My grandson has a runny nose, do you think I should take him to a doctor?’, I replied with something like, ‘If he’s fine, meaning he can eat and he doesn’t have a fever, [he will] be alright’, to just get through the situation. I’m not sure if that was a suitable answer. I don’t know if it would have been better to ask them about their symptoms in more detail or just tell them that the child is fine. When I’m afraid that asking about the symptoms in detail might take too long, I sometimes just let it go. […] If I suddenly switch to [being] a doctor and ask for details, they might [be thinking] that they didn’t want me to do that. […] I guess they were talking to me about it since I’m a doctor in the town’s clinic, [but] I wasn’t sure if I should put on a serious face as a doctor or respond like a trustworthy mother of a child.

She constructed the interaction with the child’s grandmother as a situation in which her identity could be either professional or personal, reflecting her relationship with that person, depending on her forthcoming behaviours. Such a divergent and transitory feature was plotted as an ambiguity that had posed an implicit but significant question: ‘which self should I be now?’ Here, identity development became a process of exploring and finding a suitable identity to solve the ambiguity presented by the situation at hand and an attempt to establish it with a variety of strategies. This ambiguity about and specification of one’s identities were articulated as aspects requiring one to ‘adjust(s) the misalignment’ by another participant.

When you meet someone outside, you don’t meet them as a doctor. You might meet him as ‘my son’s father’ or ‘my father’s son’. When you meet someone inside, you meet them as a doctor. When it’s the other way around, it becomes challenging. I have to adjust when I go outside as a parent and they come to me as a doctor, or when I act as a doctor inside and they come to me as a son. I would call it ‘balancing’ when I behave and move in such a way that adjusts the misalignment […].

Significant strategies to achieve the ideal identity state involved reconciling identities and rural situations. There appeared to be differences in the participants’ familiarity with such strategies, depending on their upbringing and duration of experience in rural practice. Physicians like Leona, who had grown up in relatively urban areas and had been working for a short time in rural areas, expressed uncertainty in such practices. In contrast, some who had grown up in smaller populations or had been working in rural settings for a longer period were more explicit about their practices and had become proficient in their identity work. For example, Chizuo used the metaphor of ‘put on an inner white coat’ when he explained how he deals with solicitations of medical advice in non-clinical settings. This is a succinct expression of the proficiency of how he reconciles his identity and rural life situations, which he acquired through his rural experiences.

When I’m in my hometown, neighbours or parents of my children’s classmates sometimes ask me for medical advice, and of course, I respond to them. Well, I guess it’s not so different [from this place]. In the past, I would have thought, ‘What?’ But lately I’ve rethought what I should do. When I respond to a request for medical advice, it’s like putting on an inner white coat in a flash. But I generally take it off. I don’t act like a physician all the time.

Strategies to address the fluidity emanating from the multi-facetedness of identity included the *arrangement* of the circumstances and *specification* of one’s identities. *Arrangement* refers to constructing a ‘clinical’ or ‘non-clinical’ setting to mediate their circumstances. *Specification* refers to articulating which identity one is assuming amidst their multi-faceted identities, which includes the physician’s decision to respond (or cautiously reject) the expectations of their neighbours or patients. These identity formation strategies in this narrative focus on the adjustive process through which their identity and situation are reconciled to settle the divergence and clarify the ambiguity. Enishi, who was born and raised in a very small population and had worked as a physician in the area for years, described how he arranged the situation to reconcile his identity and the situations he found himself in.

When I go out as a father and I’m playing in the neighbourhood park, it’s a little tricky for me if someone asks me for advice about medication. But since I’m a father here, I can call out [my] child’s name and say, ‘Oh, yes, that’s right, well, since Dr M (his colleague) is still working at the outpatient clinic today, let’s check whether we can consult with him’. That’s how I achieve a balance. Suppose you have a private individual side and a doctor side, although it’s not so simply binary, it’s okay if you can act as the former outside and the latter inside the office. But if communication is brought up that distorts the alignment, I try to gently align them.

Enishi resolved the misalignment using a combination of strategies including arranging the situation (showing that he is with his child at that moment) and implying his on-the-spot personal identity with subtle words (calling their child’s name). Some participants applied these strategies during clinical-hour interactions if necessary. Enishi expressed how he explicitly specified that ‘he is a physician’ when his identity as a community member is likely to intersect with the patient’s agenda.

During Mr Minami’s consultation, he wanted to make a secret confession. At that time, I assured him that although we lived in the same area, he was currently in the doctor’s office and that the information he shared here would never be passed on to anyone else. This gave him the confidence to proceed. At the next visit, I supported him by asking him if he felt burdened after the conversation, while also assuring him that he shared something very important. He responded that it made him feel better and [that] he [didn’t] think I would disclose the secret in the community, so it was totally alright.

These strategies are inherently temporal and intermittent since one’s identities occasionally and repetitively become fluid and the ambiguity therein recurs due to a constantly transitioning rural situation. Thus, the identity formation in this narrative is constructed as an intermittent fixing of situation-dependent identities amidst this recurrent ambiguity, which stems from the multi-faceted relationships with local members.

### Maintaining multi-faceted identities for flexibility

The fourth identity narrative was a process of embracing the generativity of the fluidity stemming from multi-faceted identities and avoiding their convergence to cope with emerging relationships in the rural community. The fluidity was constructed as a flexibility rather than an ambiguity and its preservation or even construction was regarded as ideal.

This narrative embraced the value of maintaining fluid positions in the local community by deliberately occupying a place where their labelling is a ‘local citizen’ and a ‘physician’. The focus on the flexibility of identity formation fluidity is mentioned by Enishi who has worked for several years in his rural hometown.

Refusing or avoiding conversation does not mean keeping a proper distance. Instead, the closer you get to them, the more you talk to them, the more appropriately you can keep your distance. When you meet someone in the park, at the pool, or at a festival, it’s better to have a small, casual conversation. […] For example, there was an old lady in end-of-life care who was getting worse and worse, and everyone around her was saying that her son probably couldn’t take care of her at home. When I was cycling with my children, the old man (her son) was working hard in the rice paddies. I wanted to ask him about his mother’s condition and how he was going to make decisions in the future, but [instead, I greeted] him saying ‘It’s hard work in the rice fields’. He said, ‘Hi, what are you doing, doctor?’ I said, ‘Well, I’m taking a walk with my child, and I want to go home, but she says she still [wants to go on]’. It’s better to have a conversation that you can have on the spot, so that you will be able to step in when you need to and you will be able to back off when the person would step in [and you need to back off]. It becomes easier to adjust in that way.

He described identity formation for rural physicians as constructing and maintaining several connective facets with the locals since there is more flexibility in managing complex negotiations based on clinical tasks and local members’ expectations. The multi-facetedness and consequent fluidity of identities are constructed as a source of capability, which is a prerequisite for interactions with stakeholders, by providing repertoires of identities. This point is elaborated in Enishi’s excerpt below.

My opinion is that a person has a range of aspects that enable potential connection with others, and the more variable aspects a physician has, the more diverse patients they can make relationships with. Then, ‘the local’ or ‘physician’ identities are only aspects of the person. You can become flexible if you are aware that you have a range of other aspects. That is, if you notice only two aspects, it is harder to fit in various situations, but if you are aware of 20 aspects, you can shift the conversation from daily chats to serious talk more flexibly.

Thus, the fluidity that exists between the multi-faceted identities was constructed as a feature of the ideal identity rather than a problem to solve. Accordingly, the strategies aimed for the preservation of this fluidity. For example, in the context of the community-oriented health promotion project led by local initiatives, Chizuo deliberately placed himself as an ‘observer’ who is not identical to the local members, while as a project team member, he introduced the activity to the health care staff at his clinic. He strived to position himself as an outsider as well as a participant in the local project team and the project representative in his clinic. This can be considered *liminalisation*, or maintaining a position betwixt and between the local community and the community of health professionals. Similarly, Enishi explains how he attempts to construct the multiplicity of his identities in the context of community events by metaphorically mentioning his approach to ‘decrease the density of the physician aspect’, as shown below.

When I go to citizens’ round-table meetings or events by the council of social welfare, I go there as a citizen even though I also go there as a doctor. If I’m only focused on [the] doctor aspect, I would broaden the scope of what I say by talking about child rearing or welfare issues in the community, not just medical topics. That’s how I’ve been able to reduce the density [of my physician aspect]. However, I don’t disappoint them; as a doctor, I can tell them that there is a problem with the aging of the population and that there are patients with dementia and so on since there is a role they expect me to play in this regard.

These acts of narrating from multiple identities, or *polyphonisation*, constitute a specific strategy in this narrative. Another participant, Akari, described how she used polyphonisation during her clinical encounter after she left rural practice and moved to an urban clinic, to reconcile her private and professional identities associated with patients. After leaving rural practice, she gave birth while working in an urban area and received baby gifts from some of her patients. Akari realised from her rural experience that sharing both her private and professional identities with her patients was her approach to sustainability in a given region. By returning gifts to her patients, she tried to express her multiplicity at the end of clinical encounters with them, which is narrated as follows.

I gave birth in Kotani, and there were some patients who [had given] me baby gifts, and I [wanted] to give them a gift in return. […] That [situation] is different from [that between] a medical doctor and a patient in [a] medical care [context], isn’t it? Well, [in this case] I was the one who had given birth to a child, and the patients were the ones who congratulated me and gave me a gift. So it’s similar to Mutsuki Island, where there is a relationship between the medical staff and the patients, plus something more. [Anyway], I wanted to give something in return, not as a doctor, but as someone who is giving back. So I drew a line from the clinical consultation and said that from here on, I will talk as a person [who] had received a baby gift.^1^

Akari’s attempt in this context can be understood as constructing an identity of a gift recipient by compartmentalising the conversation in the examination room where her identity tends to be centred on the physician identity. The common question in such identity narratives is based on the ‘and’ perspective (how can I maintain both identities − A and B − in this situation?). In these ways, positioning one’s identity in the place of betwixt and between (*liminalisation*) or embedding one’s multiple identities into the interactions (*polyphonisation*) were understood as strategies to maintain multiplicity for the consequent divergence and generativity.

### Combining different identity narratives

Some participants used different identity narratives depending on their experiences or the events they encountered during their rural placement. Although it is not our intention here to comprehensively describe the variety of combinations, our results suggest at least two patterns of combining multiple identity narratives. First, participants who used multiple identity narratives dominantly applied one identity narrative type and used another type in a subordinated tone. For example, some participants who narrated experiences mainly with the third identity narrative used the first identity narrative to describe an event where they felt ‘distance’ was necessary. However, they minimised its significance by labelling it as an ‘exception’ or ‘transient’ and did not continue to apply the first identity narrative to other subsequent experiences. Likewise, other participants who exclusively used the first identity narrative also used the third identity narrative when talking about an experience in which a non-physician aspect happened to contribute to a positive doctor–patient relationship. While they acknowledged the potential positive aspect, they retained the distinction as is by referring to personal preferences or norms of objectivity.

Second, some participants used different identity narratives depending on the period of the rural placement. One participant explicitly provided an example of this shift. Chizuo, as mentioned above, completely changed his identity narrative structure from binary oppositive to multi-faceted as his rural experiences progressed. When he articulated this change, he described his experience of contacting and interviewing rural lay people in other districts as an eye-opening experience.

I thought I understood the local members’ thoughts very well since I was listening carefully to my patients in my practice. But when I visited rural places where I was not involved with clinical care and talked to the people there, they spoke frankly. They spoke of many things and I felt ‘Oh, this is what [they] actually think.’ […] They spoke of things I’d never heard about in the clinic. […] Even in communities where certain doctors had been rooted and practiced for many years, people often harshly condemned those doctors. […] Some of them seemed to have unreasonable expectations. But [I] realised that this was the way lay people who are not formally trained in medicine really felt after all.

Chizuo juxtaposed this experience with the term ‘accessibility’ when he reflected on this experience and the shift in his stance on out-of-clinic consultations.

After hearing them talk and reflecting on the experiences, I thought that accessibility, in many ways, was everything. Of course, it means access to medical care immediately at any time, but also the emotional distance between medical care and local people. Not only doctors but also medical care, medical institutions, health information, and so on. I realised that these things related to medical care and health were poor in terms of accessibility for various reasons. There are of course physical factors such as distance, lack of medical facilities, lack of doctors, and others but also lack of information. Even if these services are available, they are not well-provided, and the recipients don’t know how to receive them, or they don’t try [to receive them]. I strongly felt that.

Application of the term ‘accessibility’, which is often referred to as a principle of primary care, as a prioritising virtue suggests that Chizuo’s shift is not only about how he addresses out-of-clinic requests but also about his ideal of how a physician should be. The way he deployed the idea of accessibility to widespread aspects of healthcare also suggests that the virtue of accessibility affected his dominant identity narrative.

## Discussion

### Overview of principle findings

This study aimed to describe diverse identity narratives of rural physicians, focusing on their experiences of physician-patient interactions in non-clinical settings and the difficulties thereof. An overarching theme pertaining to all narratives was how rural physicians configurated their participation to their local communities and to being a physician. The participants’ identity narratives were divergent in various dimensions, from (a) fundamental presumptions regarding themselves and their local communities to (b) their ideals of and strategies for identity formation. The identity narratives were framed based on two underlying structures: binary oppositive (identity narratives (i) and (ii) in Table 2) or multi-faceted (identity narratives (iii) and (iv) in Table 2) structures. The former structure assumed that the physician and the local community contrast each other and that identity formation is a goal-oriented process, whether the goal was keeping a distance from the local community or immersing oneself in it. Narratives based on this structure tended to be justified by the norms of the community to which they aspired to belong. Identity was represented as a singular, internal, united entity. The latter structure assumed that identity formation was an adjustive process responding to constantly changing situations. Under this structure, identity construction was not guided by a clear goal but rather by situated appropriateness. Against the backdrop of the divergent assumptions on the self and the form of participation, we further explicated the divergence of identity narratives concerning what was narrated as implied ideals and strategies in the identity development context.

### Diversity of the identity narratives of rural physicians − challenging the ‘discourse of integration’

Our focus on rural physicians’ non-clinical experience has magnified the diverse identity narratives by which they positioned themselves while forming their identities amid the interplay between the norms of the medical profession and those of the local community. The theme pertaining to these narratives was the diversity regarding how they experienced, managed, and positioned multiple participatory processes between professional and local rural communities, and how the processes were narrated differently across participants. Participation in multiple communities, whether concurrently or subsequently, is accompanied by the experience of boundary crossing (Akkerman & Bakker, 2011; Hazen et al., 2018; Kluijtmans et al., 2017). The findings from this study explicate the diversity of the identity narratives regarding how the boundary was constructed, experienced, and dealt with. For the first and second narratives, it was a given, hard-and-fast line between the health professionals and local people. Whether it was something that had to be maintained (in the first narrative) or resolved (in the second narrative), these narratives were framed by an aspiration for a silo community in the PIF. This might be reflective of a persistent notion valuing self-governance in mono-professional communities and strict role boundaries in medical education (Stalmeijer & Varpio, 2021). Such a notion that values participation in a single community may lead physicians to feel as though they are positioned at the periphery of the professional community and at risk of never fully belonging to or being acknowledged as a legitimate member, both by other clinicians and local people (Akkerman & Bakker, 2011). Meanwhile, the boundary in the third and fourth narratives arose from situated interactions with stakeholders. The discourse focused on the process of habiting in the boundary between the professional community and community of local people. The process of habiting in the boundary was oriented towards maintaining productive multi-membership (Wenger-Trayner et al., 2014) rather than single participation in PIF.

The third and fourth narratives seem to be contrastive regarding how the multi-facetedness and resultant fluidity of identity were positioned. While the third represented this multi-facetedness and fluidity as an ambiguous dilemma, the fourth narrative positioned them as a source of creativity. Consequently, while the former constructed them as a transitory status that should be overcome, the latter positioned them as a feature in identity formation that should be deliberately constructed and maintained. The subsequent strategies (liminalisation and polyphonisation) aligned with the ideal position, which aimed to avoid the convergence of their identities into a mono-identity (e.g. physician identity). Some participants concurrently used the third and fourth narratives, which constitute a continuous iterative process between the convergent process (specification) and divergent process (polyphonisation). The former identity formation process seemed to be adjustive in response to the conflicting expectations of others during their rural life. The latter, that is, embracing the generativity and avoiding the convergence of their identities, seemed to be a requisite for uncertain future demands, which include everlasting negotiated processes in the rural community where either the predefined medical-profession- or local-community-based norms will not do. This affirmative sense-making and active maintenance of the multi-facetedness of identities are scarcely pointed out in existent literature regarding PIF. An exception is a study by Gordon et al. (2020) exploring physicians’ identity transition from trainee to trainer by focusing on their liminality. Their results based on the notion of ‘occupying liminality’ are similar to our findings in terms of active maintenance. However, while this ‘occupying liminality’ notion is a preparatory state to survive the transition, which suggests the progression to an ensuing identity status, the fourth narrative in our study placed the multi-facetedness of identities not as a transitory state but as an ideal status to be actively achieved and maintained.

The scarcity of multi-faceted identities as a creative and ideal feature in existent studies of PIF would reflect a biased assumption underlying the existent dominant PIF frameworks for rural physicians as well as PIF in general. A previous review finding by Parlier et al. (2018) representing a model of PIF for rural physicians, as well as the widely accepted model among professionals in general presented by Cruess et al. (2015), suggest an ‘integrated identity’ as the developed feature of identity formation. While these models do not articulate what integration is, the schemes imply that forming a coherent and homogenous whole is an identity formation ideal and multiple identities should be merged into a coherent mono-identity. These assumptions influence what type of participation is deemed appropriate and how multi-faceted identities and consequent complex participatory processes have been articulated. Participation to a monolithic, uniform (clinician’s) community would be standardised as a prototype, while more complex participations associated with multi-faceted identities are regarded as something exceptional and can be remediated to more simplified participation. This ‘discourse of integration’ and its consequent assumptions concerning PIF research are problematic. They might lead educators and learners to neglect multiple participations except for the dominant one. They might inadvertently elevate learners who experience PIF as an element of simple participation in a monolithic community and relegate learners who experience PIF as a more complicated process. In addition to threatening the legitimacy of maintaining multiple identities, they might focus only on the challenging aspect of multiple participation and conceal its creative and constructive aspects by forcing the idea that multiple identities should be ‘fixed’ to achieve a harmonised whole. As a result, they would limit the diversity of the PIF in which multiple types of participation is common, as evinced by the narratives of rural physicians in this study.

To overcome this issue, this study calls for several transitions concerning the research and education of PIF. First, multiple participations should be paid more attention and acknowledged. Research outside of the medical education field suggest that multiplicity of identities is the rule rather than the exception (Akkerman & Bruining, 2016). This can be partly accomplished if researchers and educators consciously apply theoretical perspectives which explicate the multiple participatory processes of physicians. For example, some studies of clinician-scientists (Kluijtmans et al., 2017), undergraduate teachers (van Lankveld et al., 2017), and participants of faculty development (Balmer et al., 2021) applied theoretical perspectives to illustrate how clinician-scientists and faculty members juggle multiple identities in their practice and identity development. Our finding suggests that the stance should be extended to trainees and clinicians even without additional academic or educational roles.

Second, it is necessary to challenge the ‘discourse of integration’ in which multiple aspects can be or should be ‘integrated’ or merged as a consolidated or harmonised whole. Scholars and educators should not leave ‘integration’ as an idealised position without articulation. To achieve this, we argue that it is necessary to critically examine integration as well as its underlying perspectives such as those regarding the nature of the person and identity. An assumption requiring attention is that a ‘proper’ personhood is individualistic and reflects a person who can integrate a range of identities into a coherent, consistent whole. On this point, the diverse perspectives regarding the nature of the self and identity are articulated by Hermans and Hermans-Konopka (2010), who consider the modern, postmodern, dialogical self. In addition, some studies in anthropology and cultural psychology have articulated how the conception of personhood and identity are diverse across social contexts. One classic dimension is the individual-dividual axis (Hess, 2006; Smith, 2012). While the former suggests that a person should constitute a harmonised whole and be indivisible, the latter suggests that a person embodies multiple personas that potentially conflict or contradict each other. Although such a binary viewpoint is too simplistic, these contrasting perspectives can be a primer to articulate potential diversity regarding what a feature of a person is and should be, as well as how the integration and multiplicity of identities are positioned in the discourse of PIF. For example, the idealised status of integration seems to derive from an assumption that the self cannot be divisible and multiplicity should be merged and solved for a person to mature. However, such a perspective is essentially a construction based on particular social contexts rather than a universal truth. Although several PIF studies have applied the perspective of acknowledging the dividual aspect of identity and personhood (Dornan et al., 2015; van Lankveld et al., 2017), the dominance of this perspective on integration has rarely been explored; in the context of the current study, this might lead to relegating the multi-facetedness of the physician identity, particularly when one’s career is heavily influenced by participation in different non-clinical communities.

### Limitations

This study has several limitations. First, it was based on interviews, relying on participants’ perspectives and recollections of their experiences, and was not a field study that took place in situ. Accordingly, the findings might have been influenced by the relationship between the participant and the researcher, both of whom were rural physicians. For example, the narratives that differentiated physicians and local communities might reflect the performative act of the participants justifying their status rather than speaking from actual experience as rural physicians in the community. A focus on the language used in daily conversation would be appropriate to overcome this limitation (Monrouxe, 2010). Second, since the aim of this study was to illustrate rural physicians’ divergent identity narratives, it did not articulate why and how divergences evolved and what factors influenced the construction of particular identity narratives. Moreover, this study is based on a retrospective identity development narrative, which makes it difficult to identify the factors that contributed to participants’ construction of particular identity narratives and the factors that caused them to change from one narrative to another. Although some of our study findings suggest how identity narratives shift, further studies are necessary to identify the kind of rural experiences that might promote or hinder changes in identity narratives and what educational approach is required to understand and support the PIF.

Another limitation of this study is the transferability of the findings. The participants of the study were Japanese rural physicians with limited diversity regarding their familial, gender, and ethnic contexts. Particularly, an examination of rural physicians with more diverse familial contexts would be necessary since such diversity might significantly influence the diversity of their experience in non-clinical settings (Hancock et al., 2009). Finally, this study is based on data collected between 2015 and 2017. As the context surrounding undergraduate and postgraduate medical education as well as wider social situations have changed significantly since then, it is vital that current clinical teachers and researchers interpret the present findings cautiously.

### Implications for future education and research

This study, even with the aforementioned limitations, has significant implications for future educational practice in rural medicine as well as medical education in general. First, reflection on non-clinical patient interactions should be given more attention in medical education. Such attempts would open an educational opportunity to explore and support individual learners’ PIF by helping them to express their conflict and congruence between internal values and daily experiences that are otherwise less likely to be discussed. Such opportunities would be useful for avoiding the subordination and exclusion of significant rural experiences in PIF discourse and for ensuring a more inclusive medical curriculum.

For clinical teachers and curriculum developers who are involved in supporting rural physicians’ and prospective medical students’ PIF, the result of this study would provide a preliminary framework. It can help them understand rural physicians’ PIF and related issues, and provide a useful lens to explore learners’ perspectives on themselves, the rural community, and their relationships, which form the paradigmatic ground of identity formation. However, it was not our intention to classify individuals’ PIF into four narratives. Such a diagnostic classification can risk learners attempting to fit into rigid categories and hinder flexible changes responding to forthcoming local situations. Instead, clinical teachers should utilise the findings to contemplate their assumptions and values and those of their learners concerning PIF to become cognisant of what aspects of PIF can be divergent and which have been excluded. Our results demonstrated that the divergence includes not only challenges and coping strategies but also basic assumptions including what constitutes the self and the surrounding community, and the ideal identity. This application requires cultural humility where clinical teachers commit and engage in self-reflection and self-critique as lifelong learners and check the power imbalances within the dynamics of teacher–learner interactions (Tervalon & Murray- García, 1998). The reflexivity on power imbalances has been pointed out in the context of PIF, ethnicity, gender, and race (Wyatt, Balmer et al., 2021). Our study suggests that the relevance of reflexivity is not only related to particular social attributes but also to particular medical specialties.

Another implication for clinical teachers and curriculum developers is the necessity of paying attention to their learners’ multiple participatory processes. Unfortunately, medical curricula have rarely harnessed learning from a theoretical perspective for multiple participatory processes such as a boundary crossing perspective, although it has been suggested in other areas such as inter-professional education and raising physician-scientists. This may be due to clinicians tending to be less accustomed to working beyond their current roles and identities (Hofmann & Vermunt, 2021). Some scholars suggest that existing notions regarding role boundaries, education, and training silos and related epistemic organisational boundaries are barriers for promoting this kind of learning (Stalmeijer & Varpio, 2021). By focusing on PIF with theoretical perspectives explicating multiple participatory processes, future medical curricula would be able to fully articulate PIF experiences as well as harness the missed opportunities for learning concerning multiple participatory processes.

Finally, curriculum developers and researchers of PIF should reflexively examine the limitations of the current PIF model, including the ‘discourse of integration’, in describing the goal of medical education. They should be clear on what they mean by ‘integration’ and how they position it in the context of supporting and researching PIF. For instance, Syed and Mclean (2016) have pointed out that despite the multiple dimensions of integration, researchers tend to focus on one aspect of it. We propose that scholars be cautious about the potential multiplicity of integration as a concept and critically question its ubiquity as an ideal state across various sociocultural contexts. Such explicit articulation would expose possible variations regarding ideal PIF as well as how that ideal can be achieved. The identity narratives provided in this study could become the primers of changing PIF narratives in medical education to achieve inclusive support.

## Conclusion

In this research, we have delineated the differences in rural physicians’ identity narratives and their underlying PIF processes. Our findings could inform individual learners, clinical teachers, and curriculum developers about the potential diversity of the rural physicians’ PIF and help them to become reflexive regarding their range of assumptions regarding PIF. The findings will also be meaningful in supporting individual rural physicians as well as making future medical curricula inclusive. In addition, our study urges the researchers exploring PIF to examine their unexposed assumptions regarding rural physicians’ PIF as well as those of physicians in general. Such assumptions include the representation of single participation as a mainstream process of physicians’ PIF and the ‘discourse of integration’, which set integration as an implicit ideal status. Future educational practice and research should proceed based on the critical examination of these assumptions and their consequences on individual learners beyond rural physicians and curriculum design. By doing so, academic and educational discourse regarding PIF could help the careers of rural and other physicians flourish.

## Electronic supplementary material

Below is the link to the electronic supplementary material.

Supplementary Material 1

## Data Availability

No datasets were generated or analysed during the current study.

## Footnotes

1 In Japan, when people receive gifts to celebrate the birth of a child, they traditionally have to give a gift in return to show appreciation for the original gift.

## References

[CR1] Akkerman, S., & Bakker, A. (2011). Boundary crossing and boundary objects. *Review of Educational Research*, *81*(2), 132–169. 10.3102/0034654311404435.

[CR2] Akkerman, S., & Bruining, T. (2016). Multilevel boundary crossing in a professional development school partnership. *Journal of the Learning Sciences*, *25*(2), 240–284. 10.1080/10508406.2016.1147448.

[CR3] Balmer, D. F., Rosenblatt, S., & Boyer, D. (2021). Navigating landscapes of practice: A longitudinal qualitative study of physicians in medical education. *Medical Education*, *55*(10), 1205–1213. 10.1111/medu.14572. 34060657 10.1111/medu.14572

[CR4] Bleakley, A. (2005). Stories as data, data as stories: Making sense of narrative inquiry in clinical education. *Medical Education*, *39*(5), 534–540. 10.1111/j.1365-2929.2005.02126.x. 15842721 10.1111/j.1365-2929.2005.02126.x

[CR5] Brooks, K. D., Eley, D. S., Pratt, R., & Zink, T. (2012). Management of professional boundaries in rural practice. *Academic Medicine: Journal of the Association of American Medical Colleges*, *87*(8), 1091–1095. 10.1097/ACM.0b013e31825ccbc8. 22722347 10.1097/ACM.0b013e31825ccbc8

[CR6] Clandinin, D. J., Cave, M. T., & Berendonk, C. (2017). Narrative inquiry: A relational research methodology for medical education. *Medical Education*, *51*(1), 89–96. 10.1111/medu.13136. 27807868 10.1111/medu.13136

[CR7] Crampton, P. E. S., & Afzali, Y. (2021). Professional identity formation, intersectionality and equity in medical education. *Medical Education*, *55*(2), 140–142. 10.1111/medu.14415. 33179338 10.1111/medu.14415

[CR8] Cruess, R. L., Cruess, S. R., Boudreau, J. D., Snell, L., & Steinert, Y. (2015). A schematic representation of the professional identity formation and socialization of medical students and residents: A guide for medical educators. *Academic Medicine: Journal of the Association of American Medical Colleges*, *90*(6), 718–725. 10.1097/ACM.0000000000000700. 25785682 10.1097/ACM.0000000000000700

[CR9] Dornan, T., Pearson, E., Carson, P., Helmich, E., & Bundy, C. (2015). Emotions and identity in the figured world of becoming a doctor. *Medical Education*, *49*(2), 174–185. 25626748 10.1111/medu.12587

[CR10] Gingerich, A., van Volkenburg, K., Maurice, S., Simpson, C., & Roots, R. (2021). Urban ideals and rural realities: Physiotherapists navigating paradox in overlapping roles. *Medical Education*, *55*(10), 1183–1193. 10.1111/medu.14476. 33617663 10.1111/medu.14476

[CR11] Gordon, L., Rees, C. E., & Jindal-Snape, D. (2020). Doctors’ identity transitions: Choosing to occupy a state of ‘betwixt and between’. *Medical Education*, *54*(11), 1006–1018. 10.1111/medu.14219. 32402133 10.1111/medu.14219

[CR12] Hancock, C., Steinbach, A., Nesbitt, T. S., Adler, S. R., & Auerswald, C. L. (2009). Why doctors choose small towns: A developmental model of rural physician recruitment and retention. *Social Science & Medicine*, *69*(9), 1368–1376. 10.1016/j.socscimed.2009.08.002. 19747755 10.1016/j.socscimed.2009.08.002

[CR13] Hazen, A. C. M., de Groot, E., de Bont, A. A., de Vocht, S., de Gier, J. J., Bouvy, M. L., de Wit, N. J., & Zwart, D. L. M. (2018). Learning through boundary crossing: Professional identity formation of pharmacists transitioning to general practice in the Netherlands. *Academic Medicine*, *93*(10), 1531–1538. 10.1097/ACM.0000000000002180. 29465448 10.1097/ACM.0000000000002180

[CR15] Hermans, H. J. M. & Hermans-Konopka, A. (2010). Dialogical self theory: Positioning and counter-positioning in a globalizing society. Cambridge University Press.

[CR14] Hess, S. (2006). Strathern’s melanesian ‘dividual’ and the christian ‘individual’: A perspective from Vanua Lava. *Vanuatu Oceania*, *76*(3), 285–296. 10.1002/j.1834-4461.2006.tb03058.x.

[CR16] Hofmann, R., & Vermunt, J. D. (2021). Professional learning, organisational change and clinical leadership development outcomes. *Medical Education*, *55*(2), 252–265. 10.1111/medu.14343. 32776364 10.1111/medu.14343

[CR17] Holland, D., Lachicotte, W. Jr., Skinner, D., & Cain, C. (1998). *Identity and agency in cultural worlds*. Harvard University Press.

[CR18] Inoue, K., Hirayama, Y., & Igarashi, M. (1997). A medical school for rural areas. *Medical Education*, *31*(6), 430–434. 10.1046/j.1365-2923.1997.00699.x. 9463645 10.1046/j.1365-2923.1997.00699.x

[CR19] Jarvis-Selinger, S., Pratt, D. D., & Regehr, G. (2012). Competency is not enough: Integrating identity formation into the medical education discourse. *Academic Medicine: Journal of the Association of American Medical Colleges*, *87*(9), 1185–1190. 10.1097/ACM.0b013e3182604968. 22836834 10.1097/ACM.0b013e3182604968

[CR20] Kato, D., Ryu, H., Matsumoto, T., Abe, K., Kaneko, M., Ko, M., Irving, G., Ramsay, R., & Kondo, M. (2019). Building primary care in Japan: Literature review. *Journal of General and Family Medicine*, *20*(5), 170–179. 10.1002/jgf2.252. 31516802 10.1002/jgf2.252 PMC6732569

[CR21] Kluijtmans, M., de Haan, E., Akkerman, S., & van Tartwijk, J. (2017). Professional identity in clinician-scientists: Brokers between care and science. *Medical Education*, *51*(6), 645–655. 10.1111/medu.13241. 28247420 10.1111/medu.13241 PMC5434929

[CR22] Monrouxe, L. V. (2010). Identity, identification and medical education: Why should we care? *Medical Education*, *44*(1), 40–49. 10.1111/j.1365-2923.2009.03440.x. 20078755 10.1111/j.1365-2923.2009.03440.x

[CR23] Monrouxe, L. V., & Rees, C. E. (2015). Theoretical perspectives on identity: Researching identities in healthcare education. In J. Cleland, & J. S. Durning (Eds.), *Researching medical education* (pp. 129–140). Wiley.

[CR24] Parlier, A. B., Galvin, S. L., Thach, S., Kruidenier, D., & Fagan, E. B. (2018). The road to rural primary care: A narrative review of factors that help develop, recruit, and retain rural primary care physicians. *Academic Medicine: Journal of the Association of American Medical Colleges*, *93*(1), 130–140. 10.1097/ACM.0000000000001839. 28767498 10.1097/ACM.0000000000001839

[CR25] Penuel, W. R., & Wertsch, J. V. (1995). Vygotsky and identity formation: A sociocultural approach. *Educational Psychologist*, *30*(2), 83–92. 10.1207/s15326985ep3002_5.

[CR27] Sam, D. L., & Berry, J. W. (2010). Acculturation: When individuals and groups of different cultural backgrounds meet. *Perspectives on Psychological Science*, *5*(4), 472–481. 10.1177/1745691610373075. 26162193 10.1177/1745691610373075

[CR28] Scopelliti, J., Judd, F., Grigg, M., Hodgins, G., Fraser, C., Hulbert, C., Endacott, R., & Wood, A. (2004). Dual relationships in mental health practice: Issues for clinicians in rural settings. *The Australian and New Zealand Journal of Psychiatry*, *38*(11–12), 953–959. 10.1080/j.1440-1614.2004.01486.x. 15555031 10.1080/j.1440-1614.2004.01486.x

[CR29] Smith, K. (2012). From dividual and individual selves to porous subjects. *The Australian Journal of Anthropology*, *23*(1), 50–64. 10.1111/j.1757-6547.2012.00167.x.

[CR30] Stalmeijer, R. E., & Varpio, L. (2021). The wolf you feed: Challenging intraprofessional workplace-based education norms. *Medical Education*, *55*(8), 894–902. 10.1111/medu.14520. 33651450 10.1111/medu.14520 PMC8359828

[CR31] Syed, M., & McLean, K. C. (2016). Understanding identity integration: Theoretical, methodological, and applied issues. *Journal of Adolescence*, *47*, 109–118. 10.1016/j.adolescence.2015.09.005. 26522883 10.1016/j.adolescence.2015.09.005

[CR32] Taves, A., Asprem, E., & Ihm, E. (2018). Psychology, meaning making, and the study of worldviews: Beyond religion and non-religion. *Psychology of Religion and Spirituality*, *10*(3), 207–217. 10.1037/rel0000201.

[CR33] Tervalon, M., & Murray-García, J. (1998). Cultural humility versus cultural competence: A critical distinction in defining physician training outcomes in multicultural education. *Journal of Health Care for the Poor and Underserved*, *9*(2), 117–125. 10.1353/hpu.2010.0233. 10073197 10.1353/hpu.2010.0233

[CR34] Thach, S., Hodge, B., Cox, M., Parlier-Ahmad, A., & Galvin, S. (2018). Cultivating country doctors: Preparing learners for rural life and community leadership. *Family Medicine*, *50*(9), 685–690. 10.22454/FamMed.2018.972692. 30307586 10.22454/FamMed.2018.972692

[CR35] van Lankveld, T., Schoonenboom, J., Kusurkar, R. A., Volman, M., Beishuizen, J., & Croiset, G. (2017). Integrating the teaching role into one’s identity: A qualitative study of beginning undergraduate medical teachers. *Advances in Health Sciences Education*, *22*(3), 601–622. 10.1007/s10459-016-9694-5. 27318712 10.1007/s10459-016-9694-5 PMC5498609

[CR36] Volpe, R. L., Hopkins, M., Haidet, P., Wolpaw, D. R., & Adams, N. E. (2019). Is research on professional identity formation biased? Early insights from a scoping review and metasynthesis. *Medical Education*, *53*(2), 119–132. 10.1111/medu.13781. 30656747 10.1111/medu.13781

[CR37] Wenger-Trayner, E., Fenton-O’Creevy, M., Hutchinson, S., Kubiak, C., & Wenger-Trayner, B. (2014). *Learning in landscapes of practice: Boundaries, identity, and knowledgeability in practice-based learning*. Routledge.

[CR38] Woloschuk, W., Crutcher, R., & Szafran, O. (2005). Preparedness for rural community leadership and its impact on practice location of family medicine graduates. *The Australian Journal of Rural Health*, *13*(1), 3–7. 10.1111/j.1440-1854.2004.00637.x. 15720307 10.1111/j.1440-1854.2004.00637.x

[CR39] Wyatt, T. R., Rockich-Winston, N., Taylor, T. R., & White, D. (2020). What does context have to do with anything? A study of professional identity formation in physician-trainees considered underrepresented in medicine. *Academic Medicine: Journal of the Association of American Medical Colleges*, *95*(10), 1587–1593. 10.1097/ACM.0000000000003192. 32079956 10.1097/ACM.0000000000003192

[CR40] Wyatt, T. R., Balmer, D., Rockich-Winston, N., Chow, C. J., Richards, J., & Zaidi, Z. (2021a). Whispers and shadows’: A critical review of the professional identity literature with respect to minority physicians. *Medical Education*, *55*(2), 148–158. 33448459 10.1111/medu.14295

[CR41] Wyatt, T. R., Rockich-Winston, N., White, D., & Taylor, T. R. (2021). Changing the narrative: A study on professional identity formation among Black/African American physicians in the U.S. *Advances in Health Sciences Education*, *26*(1), 183–198. 10.1007/s10459-020-09978-7. 32572728 10.1007/s10459-020-09978-7

[CR42] Zur, O. (2006). Therapeutic boundaries and dual relationships in rural practice: Ethical, clinical and standard of care considerations. *Journal of Rural and Community Psychology*, *9*(1), 1–40.

