# Supplementary material for: An “integration” of professional identity formation among rural physicians experiencing an interplay between their professional and personal identities
Source: Adv Health Sci Educ Theory Pract. 2024 May 13;30(1):125–49. doi: 10.1007/s10459-024-10337-z (PMC11926048; doi:10.1007/s10459-024-10337-z)
Supplement: Supplementary file 1 — Supplementary Material 1 [file 10459_2024_10337_MOESM1_ESM.docx]

Supplementary material1 Interview guide

1)Demographic question

-Could you please tell me where you lived, starting from your early years, before becoming a physician?

-What were the characteristics of the places?

2)Questions about past residences as a physician and the context which brought the participants to a specific rural area.

-Please tell me where you worked as a physician thus far.

-Could you tell me the circumstances and contexts that led you to the A region?

3)Questions about participants’ daily life during placement to a specific location

-During your placement in the area, where were you residing?

-Tell me about your impressions of the region you lived in (you are living in).

4)Daily life during non-duty hours and contact with patients in a non-clinical setting.

-How did you spend your time during weekends and before/after clinic hours?

-In such moments, what kind of interactions or topics did you have with patients or residents?

-How did you handle discussions related to medical matters when they arose outside of clinic hours?

-How did you feel about engaging with patients outside of medical consultations?

-What do these interactions with patients outside of medical consultations mean to you as a physician?

-Have you ever felt a sense of duty or responsibility as a member of the community, such as ‘I should behave in a certain way’?

5)Professionalism as a physician

-Please share your perspective on what a doctor should be like, considering your experience in the local community.

6)Interaction between medical professionalism and norms of the local community

-There may be situations where the responsibilities of being a local member would conflict or are difficult to reconcile with the responsibilities of being a physician. Can you recall any such instances?

→How did you handle these situations?

→What were your personal thoughts regarding how to deal with such conflicts?

(Refer back to the earlier interview contents if they are related or there seems to be a conflict.)

-Earlier, you mentioned XXX, but it seems there is a difference from what you're discussing now. Could you elaborate on this?

6)Interaction with patients in non-clinical settings and its influence on the physician-patient relationship

-Have the interactions you mentioned earlier from your daily life ever influenced your behaviour to the patients during clinical encounters?

→Please provide specific examples.

-How did it affect your thoughts and actions during clinical encounters?

7)Debriefing

- Is there anything you would like to add or any recollections that have come to mind?
